# Tissue‐based quantitative proteomics to screen and identify the potential biomarkers for early recurrence/metastasis of esophageal squamous cell carcinoma

**DOI:** 10.1002/cam4.1463

**Published:** 2018-04-23

**Authors:** Xu‐Wei Cai, Wei‐Wei Yu, Wen Yu, Qin Zhang, Wen Feng, Mi‐Na Liu, Meng‐Hong Sun, Jia‐Qing Xiang, Ya‐Wei Zhang, Xiao‐Long Fu

**Affiliations:** ^1^ Department of Radiation Oncology Shanghai Chest Hospital Shanghai Jiao Tong University Shanghai China; ^2^ Department of Radiation Oncology Fudan University Shanghai Cancer Center Shanghai China; ^3^ Department of Radiation Oncology Shanghai Jiao Tong University Affiliated Sixth People's Hospital Shanghai China; ^4^ Department of Pathology Fudan University Shanghai Cancer Center Shanghai China; ^5^ Department of Thoracic Surgery Fudan University Shanghai Cancer Center Shanghai China

**Keywords:** Esophageal squamous cell carcinoma, metastasis, proteomic, recurrence, tissue microarray

## Abstract

Esophageal squamous cell carcinoma (ESCC) is the eighth cause of cancer‐related deaths worldwide. To screen potential biomarkers associated with early recurrence/metastasis (R/M) of ESCC patients after radical resection, ESCC patients were analyzed by a comparative proteomics analysis using iTRAQ with RPLC‐MS to screen differential proteins among R/M groups and adjacent normal tissues. The proteins were identified by qRT‐PCR, Western blotting, and tissue microarray. The protein and mRNA expression difference of PHB2 between tumor tissues of ESCC patients and adjacent normal tissues, ESCC patients with and without metastasis, four ESCC cell lines and normal esophageal epithelial cells were inspected using immunohistochemical staining, qRT‐PCR, and Western blotting. The EC109 and TE1 cells were used to establish PHB2 knockdown cell models, and their cell proliferation and invasion ability were determined by cell counting method, Transwell^®^ assay. Thirteen proteins were selected by cutoff value of 0.67 fold for underexpression and 1.5‐fold for overexpression. Seven proteins were confirmed to be associated with R/M among the 13 proteins. The potential biomarker PHB2 for early recurrence/metastasis of ESCC was identified. PHB2 expression was related to the OS of ESCC patients (*P* = 0.032) and had high levels in the tumor tissues and human cell lines of ESCC (*P* = 0.0002). Also, the high PHB2 expression promoted the metastasis of ESCC (*P* = 0.0075), suggesting high PHB2 expression was a potential prognostic biomarker. Experiments showed that PHB2 could significantly promote the proliferation and cell invasion ability of human ESCC cell lines and the knockdown of PHB2 suppressed the phosphorylation level of AKT, as well as the expression of MMP9 and RAC1. PHB2 could predict the early metastasis of ESCC patients.

## Introduction

Esophageal cancer is the eighth of most common cause of cancer‐related deaths worldwide 1 and ranks the third of incidence among all malignant tumors and the fourth of mortality cancer related in China 2. Although esophageal cancer exists in two principal forms—esophageal squamous cell carcinoma (ESCC) and esophageal adenocarcinoma (EAC), ESCC accounts for about 90% of esophageal cancer and is particularly prevalent in many developing countries, especially in Asia 3, 4. Surgery is the most promising therapeutic strategy for resectable ESCC; however, the 5‐year survival rate after surgery alone is approximately 25% 5. Postoperative recurrence and lymph node metastasis are the primary causes of treatment failure 6, 7. Therefore, screening of suitable prognostic indicator will probably be a key to monitor early recurrence/metastasis (R/M) of ESCC and discover potential treatment strategies that might reduce R/M and prolong lifetime.

Advances in proteomics technologies have enabled us to identify ectopic protein expressions and further explore the potential biomarkers and therapeutic targets for cancers. Recently, a multiplexed quantitative proteomic labeling strategy, namely Isobaric tags for relative and absolute quantification iTRAQ method, has been widely applied to global analysis in diverse cancers including breast cancer, lung cancer, and endometrial carcinoma for discovering biomarkers 8, 9, 10. The iTRAQ method is a highly sensitive proteomic platform, which is characterized by high proteome coverage and labeling efficiency, and has no side effects on analytical or biochemical properties of the labeled proteins or peptides 11, 12. However, the iTRAQ technique is proved to be more suitable for initial biomarkers discovery, and the results would require validation through a large‐scale screening 13, 14. In our study, we presented details of proteome variation in the different ESCC tumor tissues based on an iTRAQ with reversed‐phase liquid chromatography–mass spectrometry (RPLC‐MS) approach, and further validated the observations by tissue microarray (TMA) based on large samples immunohistochemistry.

To identify biomarkers for early R/M of ESCC patients after radical resection, we conducted comprehensive iTRAQ with RPLC‐MS, qRT‐PCR, Western blot analysis, and TMA analysis, comparing tumor tissues from patients with R/M after radical resection to tumor tissues from patients without R/M in 2 years after radical resection and their adjacent normal tissue samples. We obtained a protein biomarker, prohibitin2 (PHB2), which demonstrated a great promise in early metastasis of ESCC and proved to be a significant predictor for overall survival (OS). Lastly, we also performed cell and molecular biology experiments to verify whether PHB2 could affect the biology functions of human ESCC cell lines and the development of ESCC tumor tissues, and the final results told us that PHB2 could be a new target for the diagnosis and treatment of ESCC in clinic.

## Materials and Methods

### Cell culture

Human ESCC cell lines EC109, EC9706, EC18, and TE1 were obtained from the American Type Culture Collection (ATCC), and cultured in a cell incubator at 37°C with 5% CO_2_ and RPMI‐1640 medium supplemented with 10% fetal bovine serum (FBS) and 1% penicillin/streptomycin (P/S). Cells in the logarithmic growth phase were used in subsequent experiments.

### Cell transfection

Three siRNA sequences targeted at PHB2 were designed by RNAi designer, sequence as follows: shRNA1: 3′‐TGGTGAATATCTCCCTGCGAGTGTT‐5′; shRNA 2: 3′‐CAGAGCTGAGCTTTAGCCGAGAGTA‐5′; shRNA 3: 3′‐CAGCGGGCCCAATTCTTGGTAGAAA‐5′. The siRNA sequences were inserted into PLVX vector to generate PLVX‐siRNA‐PHB2. A mixture of PLVX‐si‐RNA‐PHB2, psPAX2, and pMDG2 were cotransfected into 293T cells using lipofectamin 2000 reagent (Thermo Fisher Scientific, Inc., Waltham, MA) to produce lentivirus. Lastly, EC109 and TE1 cells were infected with the recombinant lentivirus‐transducing units and 8 *μ*g/mL polybrene (Sigma, St. Louis, MO).

### Patients and clinical samples collection

ESCC patients who had undergone surgery from Jan 2001 to Dec 2009 were evaluated, and 229 patients with three‐field lymphadenectomy were enrolled in this study. The study was approved by the Ethics Committee of Fudan University Shanghai Cancer Center and Shanghai Chest Hospital, Shanghai Jiao Tong University. The written consents were received from all participants in this study. Eligibility criteria for this study were as follows: (1) pathologically confirmed primary ESCC without neoadjuvant or adjuvant radiotherapy; (2) no distant metastasis at first visit; (3) underwent a complete surgical resection (R0); (4) a total of ≥15 removed lymph nodes (LNs); (5) no sever preoperative complications; (6) no surgical contraindication; and (7) adequate clinical information and follow‐up data. Clinical data, including tumor differentiation and T/N stage, were available for all patients. The pathologic stage of each cancer at the time of operation was identified according to the TNM system 15, and the lesion was graded histologically according to the World Health Organization classification 16.

The tumor tissues and their normal tissues were procured from patients who had been given the written informed consent. Tissues were snap‐frozen after resection and stored in liquid nitrogen with the archives. The tumor tissues of patients were divided into three groups according to the follow‐up information within 2 years after radical resection: the tumor tissues from patients without R/M (Group A); the tumor tissues from patients with recurrence (Group B); and the tumor tissues from patients with metastasis (Group C). The adjacent normal esophageal tissues from patients without R/M were assigned to Group D.

The patients were followed up every 3 months after surgery for 2 years, and every 6 months thereafter. During follow‐up, regular evaluations included medical history, physical examination, complete blood count, thoracic CT and abdominal ultrasound or CT. Other tests, such as PET‐CT, were performed as clinically indicated. Contrast‐enhanced brain MRI was performed if patients had suspicious symptoms or disease progress. Disease progression was determined by the treating physician based on available information, including clinical assessments, radiologic examination and pathology reports.

### Protein extraction

Tissue samples were sectioned and thawed on ice. The tissues were washed thrice in ice‐cold phosphate buffer (PBS), ground in liquid nitrogen and extracted with lysis buffer which containing 7 mol/L urea, 2 mol/L thiourea, 65 mmol/L dithiothreitol, 0.1 mmol/L phenylmethylsulfonyl fluoride. The tissue homogenates were centrifuged with a speed of 14,000 rpm at 4°C for 30 min. The supernatant was transferred to a fresh 1.5 mL tube and the protein concentration was quantified using the Bradford Protein Assay (Bio‐Rad Laboratories, Inc., Hercules, CA).

### iTRAQ labeling and SCX peptide fractionation

The procedures of iTRAQ Labeling were conducted according to the manufacturer's instruction (Applied Biosystems, Foster City, California, CA). Equal amounts of proteins (100 *μ*g) of each group (A–D) were precipitated with acetone at −20°C for 1 h, resuspended in 20 *μ*L dissolution buffer, and then denatured at 60°C for 1 h. Afterward, the proteins were digested with trypsin at 37°C for 16 h. After trypsin digestion, peptides were dried by vacuum centrifugation and labeled with the iTRAQ regents (Applied Biosystem iTRAQ Reagents, ABI) at room temperature for 1 h. Labeling the reference sample was randomized for each set to eliminate any potential bias that might be associated with a particular iTRAQ report tag. Then, the samples were mixed with equal amounts, and desalted with the Sep‐Pak Vac C18 cartridges and dried using a vacuum centrifuge.

Strong cation exchange (SCX) chromatography was performed using a polysulfoethyl column (2.1 mm × 100 mm, 5 *μ*m, 200 Å, The Nest Group Inc, Southborough, MA) to fractionate the mixed peptides. Firstly, the mixed peptides were desalted with Sep‐Pak Cartridge (Waters, Milford, MA) and then diluted in the loading buffer (buffer A, 10 mmol/L KH_2_PO_4_ in 25% acetonitrile, pH 2.8). Subsequently, the mixture was loaded with a flow rate of 200 *μ*L/min for 1 h using a linear binary gradient of 0–80% buffer B (10 mmol/L KH_2_PO_4_ in 25% ACN, 350 mmol/L KCl, pH 2.6) in buffer A (10 mmol/L KH_2_PO_4_ in 25% acetonitrile, pH 2.8). Eventually, a total of 20 SCX fractions were collected per iTRAQ set and the absorbance at 214 and 280 nm was monitored.

### RPLC‐MS/MS analysis

The mixed peptides, which desalted with a PepMap C18 cartridge, were further separated by LC‐20AD nano‐HPLC (Shimadzu, Kyoto, Japan) on the secondary RP analytical column (ZORBAX 300SB‐C18 column, 150 mm × 100 *μ*m 5 *μ*m, 300 Å, USA). Peptides were subsequently eluted using the gradient conditions with phase B at a flow rate of 300 *μ*L/min (5% phase B for 5 min, 35% phase B for 90 min, 80% phase B for 95 min, 80% phase B for 100 min, 5% phase B for 105 min, and 0 phase B for 120 min). Phase B was composed of 95% ACN and 0.1% formic acid.

Electrospray voltage of 2.2 kV versus the inlet of the mass spectrometer was used. A hybrid quadrupole time‐of‐flight mass spectrometer (QStar hybrid LC/MS/MS Q‐TOF, AB SCIEX, USA) was operated in data‐dependent mode to switch automatically between MS and MS/MS acquisition. MS spectra were acquired across the mass range of 400–1800 *m*/*z* in high‐resolution mode using 250 msec accumulation time per spectrum. Tandem mass spectral scanned from 100–2000 *m*/*z* in high sensitivity mode with collision‐induced dissociation (CID). The four most intense ions were chosen for fragmentation per cycle with dynamic exclusion.

The LC‐MS/MS data to identify proteins were searched against the Universal Protein Resource (UniProt). Protein identification and iTRAQ quantitation were performed with Protein Pilot software (Version 3.0, AB SCIEX, Framingham, MA) with the integrated ParagonTM search algorithm. The search parameters included iTRAQ labeling at N‐terminus and lysine residues, cysteine modification by methyl methanethiosulfonate (MMTS), and digestion by trypsin. For iTRAQ quantitation, the peptide was grouped by the ProGroup algorithm in the software to calculate the reporter peak area, error factor (EF), and *P* value. In our study, the cutoff value >1.3 was applied to identify protein; the protein must be identified by a minimum of two peptides with ≥95% confidence for quantification. The results were then exported into Microsoft Excel for manual data interpretation. Proteins were considered as differentially expressed if iTRAQ ratios were ≥1.5 or ≤0.67 in ≥50% in ESCC (group A, B, C) to adjacent normal tissues (group D). In addition, proteins must show consistent changes in both replicate samples, and have iTRAQ ratios’ *P* values <0.05 in at least one of the data set to be considered as differentially expressed. The proteins were considered to be differentially expressed in a comparison in ESCC with different status of R/M (group A, B, C) also using a cutoff value of iTRAQ ratios ≥1.5 or ≤0.67.

### Tissue microarray

The tumor regions were reviewed and determined by a pathologist. Tissue microarray (TMA) blocks containing duplicate 1.0‐mm cores from each specimen were constructed with a manual tissue microarrayer (Beecher Instruments, Sun Prairie, WI). The TMAs contained 229 cases of primary ESCC and matched normal esophagus tissues. In addition, each block contained two marker cores for TMA orientation.

TMA sections were cut 5 *μ*m thicknesses from the tissue blocks and placed on charged slides. Slides were deparaffinized in xylene, dehydrated in gradient ethanol, and pretreated in a microwave oven for 20 min at 800 W in 1 L citrate buffer (0.01 mol/L, pH 6.0) for antigen retrieval. Sections were then incubated with hydrogen peroxide (0.3% v/v) in PBS for 15 min to quench the endogenous peroxidase activity, followed by blocking with 10% fetal bovine serum in PBS to preclude nonspecific binding. Thereafter, the tissue slides were incubated with primary antibodies (1:500 dilution) overnight at 4°C. Protein expression was detected using the streptavidin–biotin complex with the Dako LSAB_ kit (DakoCytomation, Glostrup, Denmark) and diaminobenzidine as the chromogen. All procedures were carried out at room temperature unless otherwise specified. The tissue slides were washed with 0.025% Triton X 100 in PBS (0.1 mol/L, pH = 7.3) three times after each step. Finally, sections were counterstained with Mayer's hematoxylin and mounted with DPX mountant. In the negative control tissue sections, the primary antibody was replaced by isotype‐specific nonimmune mouse/rabbit IgG.

Immunoexpression of each protein was evaluated by a pathologist. Quantification in tumor sections was classified into four categories: (A) moderate to strong membrane, cytoplasmic, and nuclear staining in greater than 50% of tumor cells (4+); (B) moderate to strong cytoplasmic staining in ≥50% of either the cytoplasm or the nuclei, but not both (3+); (C) moderate cytoplasmic staining in ≥25% and ≤50% of either the cytoplasm or the nuclei, but not both (2+); (D) overall weak staining in the cytoplasm and/or nuclei (1+); and (E) no staining (0).

### Western blotting

The tissue samples from frozen sections and the EC109 and TE1 cells treated as required were further taking for Western blotting. The tissue samples and cells were lysed in extraction buffer completely, centrifuged, then the supernatant was collected and the protein concentration was determined according to BCA protocol. Dysregulated proteins in ESCC samples were screened by Western blotting. Equal amount of proteins (30 *μ*g) from each patient were separated by SDS‐PAGE and transferred to PVDF membranes. The membranes were incubated with primary antibodies, including S100A9, CRNN, PRDX1, CD99, FMNL, NCL, CLIC3, SPRR3, TXLNA, TGM2, FLNA, PHB2, FABP5, MMP9, p‐AKT, AKT, RAC1 and GADPH (all from Abcam, Cambridge, MA) overnight at 4°C, then with HRP‐conjugated secondary antibodies (1:10,000 dilution) at room temperature for 2 h. Protein expression was visualized via enhanced chemoluminescence reagent (Millipore) for 5 min, and then exposed to X‐ray film. All of the above‐mentioned experiments were independently repeated three times at least.

### Quantitative RT‐PCR

Trizol homogenization buffer (Takara Inc., Mountain View, CA) was added into the tissue samples from frozen sections to extract the total RNAs, which were then reversely transcribed into cDNAs according to the manufacture's protocol from Takara. The obtained cDNAs were amplified by qRT‐PCR for S100A9, CRNN, PRDX1, CD99, FMNL, NCL, CLIC3, SPRR3, TXLNA, TGM2, FLNA, PHB2, FABP5, and GADPH (Table 1) in a total reaction system of 20 *μ*L(including 1 *μ*L cDNA template, 0.5 *μ*L each primer, 10 *μ*L SYBR green super mix and 8 *μ*L ddH_2_O). The reaction conditions were as follows: 95°C, 3 min, 1 cycle; followed by 40 cycles of 95°C 5 sec, 62°C 30 sec and 72°C, 20 sec; final cycle 72°C, 30 sec. The relative gene expression was calculated by the Livak method (2^−ΔΔCt^) with GAPDH mRNA for normalization. The experiments were repeated three times independently at least.

**Table 1:** Primer sequences used for RT‐qPCR

Name	Forward sequence (5′‐3′)	Reverse sequence (5′‐3′)
S100A9	ACAGAGTGCAAGACGATGAC	TTCACAGAGTATTGGTGGAAGG
CRNN	ACAGTGGTTGGTGAGGAATG	TGCTGAGGAAACACTGGTATG
PRDX1	GCTTCTGTGGATTCTCACTTCT	GGGTCTGATACCAAAGGAATGT
CD99	GATGCCCTTCCTGACAATGA	CCATCAACAACAGCATCTCCTA
FMNL	AGGAGCGGTTTCAAGTCAAG	TCCAATCAGCTGCTACCTTTC
NCL	ATTGGTAGCAACTCCTGGTAAG	CACTGTCATCATCCTCCTCTTC
CLIC3	TCAAGGGCGTACCTTTCAC	GCTGTCATAGAGCAGGATGG
SPRR3	CCAGCAGAAGCAGACCTTTAC	TCCTTGGTTGTGGGAACAAATA
TXLNA	GCGAGGAGCATATCGACAAA	CTTCTGCCTCCTTTAGCATCTC
TGM2	ATCACCCACACCTACAAATACC	ATCCCTGTCTCCTCCTTCTC
FLNA	CTTCGAGGTGTACGTGGATAAG	TGGTCTTGTTGGCGATGTT
PHB2	CCAAAGACCTACAGATGGTGAA	CAACACTCGTTCCTCGTAGTC
FABP5	GGCCAAGCCAGATTGTATCA	TCTCTCCCAGGGTACAAGAAA
GADPH	GGTGTGAACCATGAGAAGTATGA	GAGTCCTTCCACGATACCAAAG

### Immunohistochemical staining

The tumor tissues and adjacent normal tissues were acquired as described above and then they were fixed using 4% paraformaldehyde (PFA) for 48 h. After that, these fixed tissues were cut into 5 *μ*m slices and placed on the glass slides. Subsequently, these slides were used to immunohistochemical staining. Lastly, these slides were observed under a microscope and three random fields were photographed.

### Cell proliferation assay

The cell counting method was performed to inspect the cell proliferation ability. The EC109 and TE1 cells transfected with the siRNA‐PHB2 or siRNA‐Control were cultured in a cell incubator at 37°C with 5% CO_2_ and RPMI‐1640 medium supplemented with 10% fetal bovine serum (FBS) and 1% penicillin/streptomycin (P/S) and then they were seeded into the 10 cm dishes in a density of 2000 cells per dish. Next, these dishes were further cultured in a cell incubator at 37°C with 5% CO_2_ for 3 days and every day, the cells in one dish for each cell line were trypsinized and collected to perform cell counting. Lastly, the results of cell counting for everyday were recorded and analyzed statistically.

### Transwell^®^ assay

The cell invasion ability of EC109 and TE1 cells transfected with the siRNA‐PHB2 or siRNA‐Control was detected by Transwell^®^ assay. Cell invasion assay was performed using the Transwell^®^ system (24 wells, 8 *μ*m pore size with polycarbonate membrane; Corning Costar, Lowell, MA). The EC109 and TE1 cells transfected with the siRNA‐PHB2 or siRNA‐Control were cultured in a cell incubator at 37°C with 5% CO_2_ and RPMI‐1640 medium supplemented with 10% fetal bovine serum (FBS) and 1% penicillin/streptomycin (P/S), and then harvested and suspended, respectively, in medium without FBS. Then, the cells were added to the upper wells of the chamber by a density of 1 × 10^5^ cells. After incubated in a cell incubator with 5% CO_2_ at 37°C for 24 h, the cells adhering to the lower surface were fixed with methanol and stained with 0.1% crystal violet. Five representative fields of each insert were randomly counted under a microscope (Olympus, Japan) and analyzed statistically.

### Statistical analyses

All data analyses were conducted with SPSS 16.0 software package (SPSS Inc., Chicago, IL). Kaplan–Meier curves were constructed to evaluate group survivals, and log‐rank test was applied to analyze the statistical significance of differences. Comparisons of over two groups utilized analysis of variance (ANOVA) with post hoc testing for further paired analysis. Wilcoxon rank sum test was used for nonparametric comparisons of TMA data. The Fisher's exact tests were used to compare categorical data. Average protein expression was calculated based on two independent WB analyses. Group B and Group C samples were compared with Group A by the Mann–Whitney two‐tailed test. Other statistical analysis was performed with two‐tailed unpaired *t*‐tests. *P < 0.05, **P < 0.01.

## Results

### Patients and protocol

**Figure 1:**
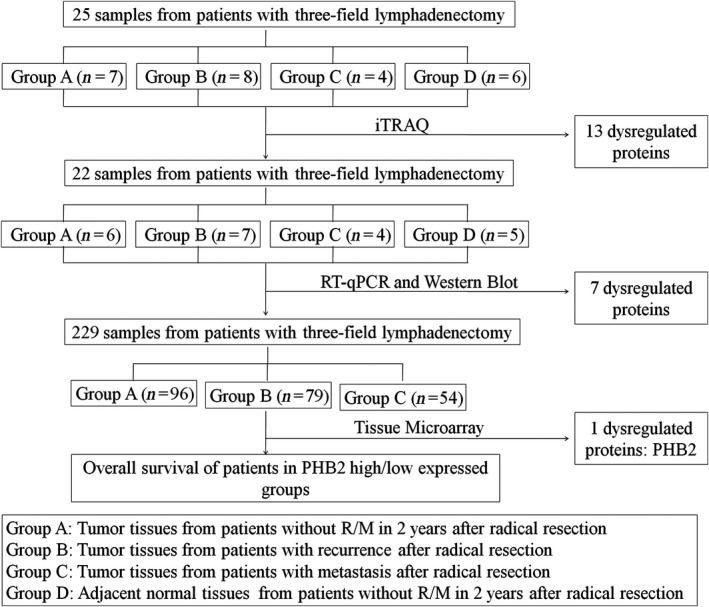
Study protocol.

Detailed research steps are showed in Figure 1. Firstly, 25 samples from ESCC patients with three‐field lymphadenectomy were enrolled in detection of 456 biomarkers associated with early R/M of ESCC patients after radical resection and were identified by applying the iTRAQ based on quantitative proteomic approach (iTRAQ‐RPLC‐MS/MS). Among them, seven tumor tissue samples from patients without R/M in 2 years after radical resection, eight tumor tissue samples from patients with recurrence after radical resection, four tumor tissue samples patients with metastasis after radical resection, and six adjacent normal tissue samples from patients without R/M were harvested, respectively. Secondly, the different proteins were identified by qRT‐PCR and Western blotting using another 22 samples, including six tumor tissue samples from patients without R/M in 2 years after radical resection, seven tumor tissue samples from patients with recurrence after radical resection, four tumor tissue samples from patients with metastasis after radical resection, and five adjacent normal tissue samples from patients without R/M. These candidate proteins were further verified by TMA in another cohort consisted of 229 ESCC patients. Finally, the prognostic significance of target protein was validated by OS of 229 ESCC patients.

### The potential biomarker PHB2 for early recurrence/metastasis of ESCC was identified

After applying a fold‐change <0.67 for down‐regulated expression and a fold‐change >1.5 for up‐regulated expression, we found four down‐regulated and two up‐regulated proteins in patients with recurrence after radical resection and another four down‐regulated and three up‐regulated proteins in patients with metastasis after radical resection, compared to the control groups (Table 2).

**Table 2:** List of 13 abnormally expressed proteins screened from proteomics

Expression pattern	Accession number	Protein	Gene symbol
Down‐regulated in tumor tissues from patients with recurrence	sp|P06702|S10A9_HUMAN	Protein S100‐A9	S100A9
	sp|Q9UBG3|CRNN_HUMAN	Cornulin	CRNN
	sp|Q06830|PRDX1_HUMAN	Peroxiredoxin‐1	PRDX1
	sp|P14209|CD99_HUMAN	CD99 antigen	CD99
Up‐regulated in tumor tissues from patients with recurrence	sp|O95466|FMNL_HUMAN	Formin‐like protein 1	FMNL
	sp|P19338|NUCL_HUMAN	Nucleolin	NCL
Down‐regulated in tumor tissues from patients with metastasis	sp|O95833|CLIC3_HUMAN	Chloride intracellular channel protein 3	CLIC3
	sp|Q9UBC9|SPRR3_HUMAN	Small proline‐rich protein 3	SPRR3
	sp|P40222|TXLNA_HUMAN	Alpha‐taxilin	TXLNA
	sp|P21980|TGM2_HUMAN	Protein‐glutamine gamma‐glutamyltransferase 2	TGM2
Up‐regulated in tumor tissues from patients with metastasis	sp|P21333|FLNA_HUMAN	Filamin‐A	FLNA
	sp|Q99623|PHB2_HUMAN	Prohibitin‐2	PHB2
	sp|Q01469|FABP5_HUMAN	Fatty acid‐binding protein	FABP5

**Figure 2:**
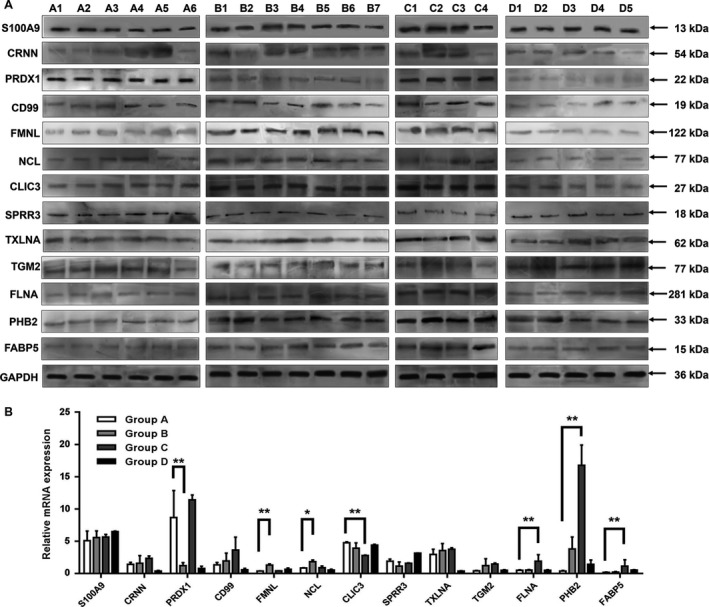
Dysregulated proteins were screened by Western blotting and qRT‐PCR. (A) Differential proteins expression in 22 clinical ESCC samples was detected by Western blotting. (B) Differential mRNA expression in 22 clinical ESCC samples was detected by qRT‐PCR. Group A: tumor tissues from patients without R/M in 2 years after radical resection; Group B: tumor tissues from patients with recurrence after radical resection; Group C: tumor tissues from patients with metastasis after radical resection; Group D: adjacent normal tissues from patients without R/M in 2 years after radical resection. Statistical analysis was performed with two‐tailed unpaired *t*‐tests. *P < 0.05, **P < 0.01.

To further elucidate the potential value of aberrantly expressed genes as biomarkers, we measured the mRNA and protein levels of the target 13 genes in another 17 tumor samples, including six tumor samples from patients without R/M in 2 years after radical resection, seven tumor samples from patients with recurrence after radical resection, four tumor samples from patients with metastasis after radical resection, and five adjacent normal tissue samples from patients without R/M. Consistent with proteomics, elevated expression of FMNL and NCL proteins and decreased expression of PRDX1 were identified in Group B compared to Group A. In addition, enhanced expression of FLNA, PHB2, and FABP5 was observed in Group C compared to Group A. In contrast with proteomics, we found that the expression of CLIC3 proteins were up‐regulated in tumor samples from patients with metastasis, while the transcripts were down‐regulated in metastasis (Fig. 2A and B).

We subsequently investigated expression of PRDX1, FMNL, NCL, CLIC3, FLNA, PHB2, and FABP5 of 229 ESCC tumor tissues by TMA. The expression levels of target protein in tumor tissues of the study cohort were scored from 0 to 4. Results showed PHB2 and CLIC3 expression were significantly up‐regulated in patients with metastasis compared with patients without R/M (*P* = 0.002 and 0.017, respectively, Table 3). Among which, PHB2 showed the consistent trend with proteomics and Western blotting, while the TMA expression of CLIC3 was consistent with the Western blotting, but contrary to proteomics results as shown in Figure 2.

**Table 3:** Results of tissue microarray in 229 patients

Group	Immunoexpressional level	PRDX1	FMNL	NCL	CLIC3	FLNA	PHB2	FABP5
A	0	2	18	4	84	4	8	9
	1	8	27	26	9	12	29	19
	2	48	40	55	1	23	44	38
	3	39	15	16	0	53	25	26
	4	6	0	0	0	4	1	2
B	0	1	17	7	72	4	5	5
	1	7	21	20	6	5	23	14
	2	32	27	36	0	14	27	32
	3	36	12	12	0	44	25	14
	4	2	0	1	0	2	1	6
C	0	1	10	2	43	1	0	1
	1	5	13	14	8	8	1	10
	2	13	19	23	6	13	26	17
	3	26	10	12	0	15	14	19
	4	3	0	1	0	1	0	1
*P* value	A vs. B	0.747	0.599	0.526	0.500	0.455	0.494	0.698
	A vs. C	0.172	0.810	0.516	0.017*	0.099	0.002**	0.139

Note: Group A: Tumor tissues from patients without R/M in 2 years after radical resection; Group B: Tumor tissues from patients with recurrence after radical resection; Group C: Tumor tissues from patients with metastasis after radical resection. Statistical analysis was performed with Wilcoxon rank sum test. *P < 0.05, **P < 0.01.

### PHB2 expression was related to the OS of ESCC patients

**Figure 3:**
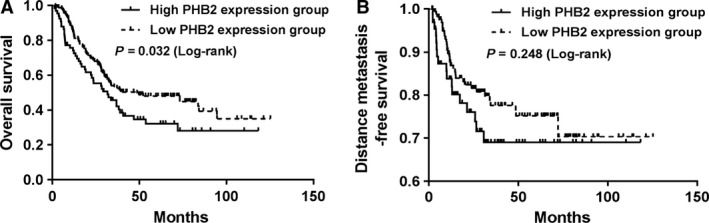
PHB2 expression was correlated with the OS of ESCC patients. (A and B) Kaplan–Meier curves of OS (A) and DMFS (B) in relation to PHB2 expression in ESCC patients with three‐field lymphadenectomy. Log‐rank *P* values are indicated within the graphs.

We classified the protein expression levels on the tissue array from weak (scored as 0~2) to strong positive (scored as 3~4). Of the 229 ESCC patients examined, PHB2 was strongly stained (scored as 3~4) in 66 cases, weakly stained (scored as 0~2) in 163 cases. Clinicopathologic characteristics of the study cohort consisted of 229 ESCC patients were summarized and compared in PHB2 strong or weak expressed groups (Table 4). The expression of PHB2 was not correlated with other clinical factors such as age, gender, tumor differentiation, T/N status and postoperative chemotherapy. However, the median survival time of high PHB2 expression group was 31.7 months, whereas the median survival time of low PHB2 expression group was 48.7 months. Although there was no statistical difference in DMFS between PHB2 high expressed and PHB2 low expressed ESCC tumor tissues (*P* = 0.248) (Fig. 3A), high levels of PHB2 expression were correlated with OS in univariate analysis (*P* = 0.032, Fig. 3B). suggesting low expression level of PHB2 could promote OS of ESCC patient.

**Table 4:** Clinicopathologic characteristics of patients in the tissue microarray assay (*n* = 229)

Characteristics	*n*	PHB2		
		Strong	Weak	*P*, strong vs. weak
Age				
<60	134	34	100	0.224
60–70	84	27	57	
>70	11	5	6	
Gender				
Female	38	9	29	0.339
Male	191	63	128	
Tumor differentiation				
I	23	8	15	0.408
II	150	39	111	
III	56	19	37	
T status				
T1	12	4	8	0.895
T2	85	22	63	
T3	104	26	78	
T4	28	8	20	
N status				
N0	84	16	68	0.242
N1	62	17	45	
N2	55	18	37	
N3	28	9	19	
Postoperative chemotherapy				
Yes	127	61	66	0.146
No	102	59	43	

### PHB2 had high expression levels in the tumor tissues and human cell lines of ESCC

**Figure 4:**
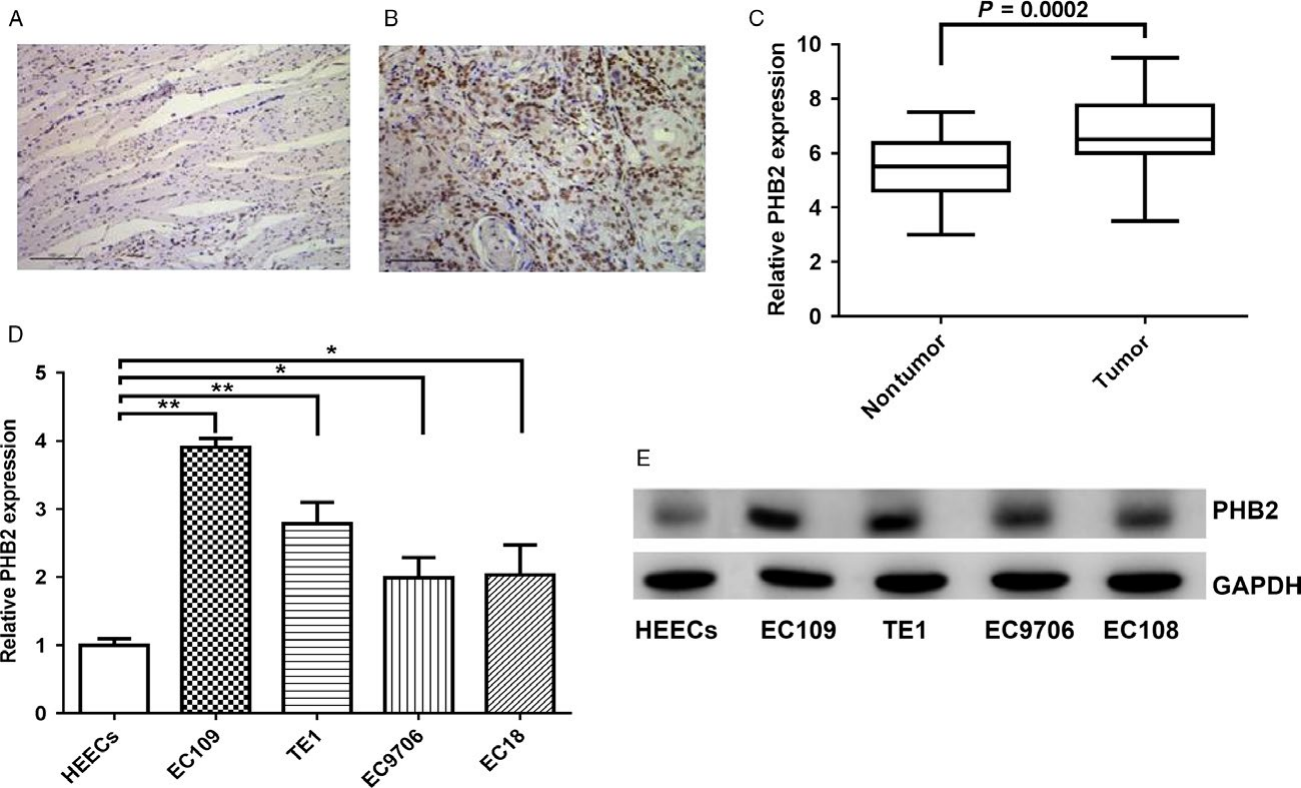
PHB2 had high expression levels in the tumor tissues and human cell lines of ESCC. (A and B) The PHB2 protein expression in the adjacent normal tissues (A) and tumor tissues (B) of ESCC was detected by immunohistochemical staining. (C) The PHB2 mRNA expression was compared between adjacent normal tissues and tumor tissues of 90 ESCC patients. (D and E) The PHB2 mRNA and protein expression in four human ESCC cell lines (EC109, TE1, EC9706, EC18) and a normal human esophageal epithelial cell line were determined by qRT‐PCR (D) and Western blotting (E). Statistical analysis was performed with two‐tailed unpaired *t*‐tests. *P < 0.05, **P < 0.01.

To investigate the role of PHB2 in the ESCC, PHB2 levels were measured in the tumor tissues of ESCC patients and four human ESCC cell lines EC109, EC9706, EC18, and TE1. Immunohistochemistry staining showed PHB2 expression in the tumor tissues of ESCC patients was significantly higher than the adjacent normal tissues (Fig. 4A and B). We determined the PHB2 mRNA and protein levels in 90 pairs of ESCC tumor tissues and their adjacent normal tissues, and the results indicated that PHB2 expression was extremely significantly higher in the ESCC tumor tissues than the adjacent normal tissues (Fig. 4C). In addition, PHB2 mRNA and protein levels were determined in four human ESCC cell lines, and the results showed PHB2 had high expression in four human ESCC cells. Therefore, these results suggested that PHB2 had high expression levels in the tumor tissues of ESCC patients and four cell lines of ESCC.

### High PHB2 expression promoted the metastasis of ESCC

**Figure 5:**
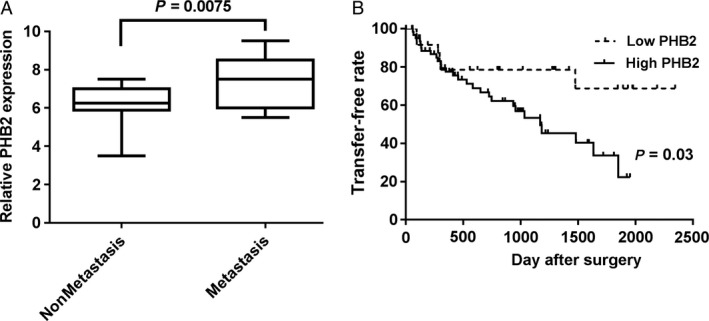
High expression of PHB2 promoted the metastasis of ESCC. The PHB2 mRNA expression was determined between metastasis and nonmetastasis in the ESCC patients. (B) The relative metastasis rate was counted among the ESCC patients with high or low PHB2 expression after surgery. Statistical analysis was performed with two‐tailed unpaired *t*‐tests.

To further investigate whether the high PHB2 expression could affect the pathological development of ESCC, we compared PHB2 expression levels in the tumor tissues of ESCC patients with or without metastasis, as well as the metastasis rate between high PHB2 expression and low PHB2 expression of the ESCC patients. Results indicated that tumor tissues of ESCC patients with metastasis had higher PHB2 expression level than the nonmetastasis patients (Fig. 5A), and ESCC patients with high PHB2 expression had higher metastasis rate than ESCC patients with low PHB2 expression after surgery (Fig. 5B). Thus, high PHB2 expression could promote the metastasis of ESCC.

### PHB2 promoted the proliferation and invasive ability of human ESCC cells

**Figure 6:**
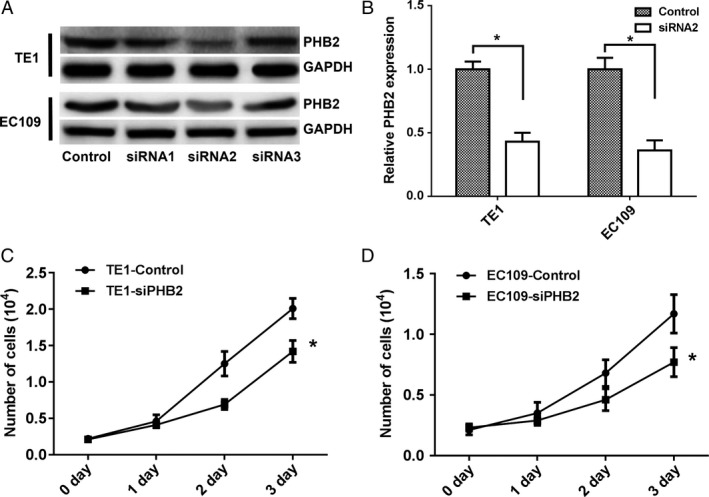
PHB2 promoted the proliferation ability of human ESCC cells. (A and B) The knockdown PHB2 expression in TE1 and EC109 cells was determined by Western blotting (A) and qRT‐PCR (B). (C and D) The proliferation ability of TE1 (C) and EC109 (D) cells transfected with siRNA‐PHB2 or siRNA‐Control were detected by cell counting method. Statistical analysis was performed with two‐tailed unpaired *t*‐tests. *P < 0.05.

**Figure 7:**
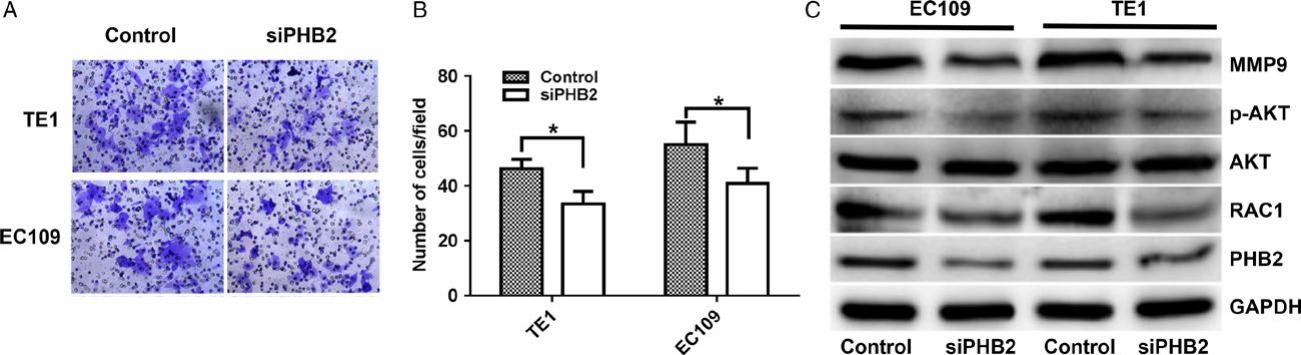
PHB2 promoted the invasion ability of human ESCC cells through activating the AKT signaling pathway. (A and B) The invasive ability was detected by the Transwell^®^ invasion assay. The two cell lines were transfected with siRNA‐PHB2 or siRNA‐Control. (C) The protein expression of p‐AKT, AKT, MMP9, RAC1 and PHB2 in the EC109 and TE1 cells was detected by Western blotting. The two cell lines transfected with siRNA‐PHB2 or siRNA‐Control. Statistical analysis was performed with two‐tailed unpaired *t*‐tests. *P < 0.05.

In order to further explore the effect of PHB2 on the biological functions of ESCC cells, we transfected three PHB2‐siRNA to knockdown in two human ESCC cell lines (EC109 and TE). Results showed that siRNA2 acquired the highest knockdown rate in both EC109 and TE1 cells (Fig. 6A), and PHB2 mRNA expression level decreased in two cell lines by PHB2‐siRNA2 knockdown (Fig. 6B). Results showed that cells containing PHB2‐siRNA significantly presented lower proliferation ability than the control cells in two cell lines (Fig. 6C and D). In addition, we detected their cell invasive abilities by Transwell^®^ invasion assays. PHB2 knockdown significantly suppressed cell invasion in two cells lines (Fig. 7A and B). And, we investigated the underlying metastasis mechanisms and found that knockdown of PHB2 obviously down‐regulated the expression levels of MMP9 and RAC1, and suppress the AKT in two cells lines (Fig. 7C), suggesting PHB2 affected metastasis *via* AKT signaling pathway. Take together, PHB2 could promote the proliferation of human ESCC cells, and their invasion ability through activating the AKT signaling pathway.

## Discussion

Despite the use of advanced surgical techniques combined with diverse adjuvant radiotherapy, chemotherapy or chemoradiotherapy, the overall 5‐year survival rate of ESCC patients is only about 40% 17, 18, 19. Thus, it is urgent to strengthen the research of discovering novel cancer diagnostic and therapeutic strategies. We performed a proteomic expression profile analysis of 25 ESCC patients after surgery resection using iTRAQ labeling technology combined with LC‐MS. After the analysis, we obtained 13 genes that could be potential candidates for the diagnosis of the early recurrence or metastasis of ESCC. With further study by qRT‐PCR, Western blotting, and TMAs, we identified PHB2 as the preferable candidate biomarker for early metastasis of ESCC, and provided evidences for its promise roles in predicting metastasis of ESCC, as well as verified that PHB2 could play an important role in regulating the biology functions of human ESCC cell lines and promoting the occurrence and development of ESCC. The integrated profiling method has been used successfully in studies on cancer outcome 20, 21, 22. However, present study using tissue‐based quantitative proteomics had identified few consistent prognostic markers for ESCC. This may be due partly to the heterogeneity of individual patients 23.

In recent years, it has attracted widespread interest that identifying important ESCC‐related biomarkers or therapeutic targets *via* the proteomic techniques. Du et al. 24 found that there were 22 dysregulated proteins in ESCCs compared to tumor‐adjacent normal tissues and that the up‐regulated expression of calreticulin and a 78 kDa glucose‐regulated protein were involved in poor prognosis through proteomic profiling. In a study by Cao et al. 25, Annexin II, kindlin 2, and myosin 9 were proved to be abnormally expressed in ESCC samples and could be significant predictors of ESCC OS. Additionally, transglutaminase 3 expression was validated in 76 primary tumor samples by IHC and found to be inversely correlated with shorter disease‐specific survival rate 26. In the other quantitative tissue proteomic studies, various proteins including *α*‐actinin 4, galectin 7, calreticulin, periostin, and the gene amplified in squamous cell carcinoma have been demonstrated to be potential biomarkers 27, 28, 29, 30, 31. According to previous reports, approximately 50% of the ESCC patients who had undergone surgery experienced postoperative recurrent or metastasis disease 32, 33. Uchikado et al. 34 showed that Slug expression in the E‐cadherin preserved tumors is related to prognosis in patients with ESCC and Slug in preserved E‐cadherin group is useful for predicting malignant properties of ESCC. Kitaichi et al. 35 suggested that loss of PAR‐3 protein expression may lead to tumor progression and subsequent lymph node metastasis in ESCC and was associated with adverse prognostic factors in ESCC. However, the biomarkers for predicting early recurrence or metastasis of ESCC patients remain to be poorly understood. In this study, we identified 13 dysregulated expression proteins by iTRAQ and then found seven candidate proteins in 13 dysregulated expression proteins by Western blotting and qPCR. The seven proteins located in cytoplasm or secreted to extracellular region, such as PRDX1, FMNL, NCL, CLIC3, FLNA, PHB2, FABP5, which could observe in the same conditions by TMAs. Eventually, protien PHB2 expression inquantitative proteomics approach, Western blotting, qRT‐PCR, and TMAs were consistant. Thus, we speculated PHB2 may be related to ESCC. PHB2, prohibitin proteins 2, is a evolutionarily conserved protein and a repressor of estrogen receptor (REA) activity or B‐cell receptor associate protein (BAP) 37, and can be found in bacteria, fungi, plants, and mammals 36. Previous studies have documented that PHB2 plays a vital role in repressing ER signaling 37, regulating cell apoptosis 38, and modulating cell cycle progression *via* the interaction with p53, E2F and pRb 39, 40. In addition, aberrant expression of PHB2 has been observed in multiple cancer cell lines and tumor tissues including breast cancer, rhabdomyosarcoma, thyroid cancer, neuroblastoma, and hepatocellular carcinoma 41, 42, 43, 44, 45. However, whether PHB2 is involved in ESCC remains to be explored. We found that PHB2 expression was related to the OS of ESCC patients and had high levels in the tumor tissues and human cell lines of ESCC. Also, the high PHB2 expression promoted the metastasis of ESCC, suggesting high PHB2 expression was a potential prognostic biomarker. Experiments showed that PHB2 could significantly promote the proliferation and cell invasive ability of human ESCC cell lines and the knockdown of PHB2 suppressed the phosphorylation level of AKT, MMP9, and RAC1, which also involved in the metastasis mechanisms of some cancer cells, which indicated that PHB2 may be associated with the postoperative metastasis of ESCC via AKT signal.

In summary, we identified and further investigated PHB2 might be considered as a potentially prognostic marker for predicting the early R/M of ESCC after radical resection and could promote proliferation and metastasis of human ESCC cells through activating AKT signaling pathway, which provides a theoretical basis for further mechanism research on ESCC R/M.

## Conflict of Interests

These authors disclose no conflicts of interests.
